# Dr. L. A. Babcock’s Silver Uterine Supporter

**Published:** 1869-07

**Authors:** 


Dr. L. A. Babcock's Silver Uterine Supporter.—We have received one of these instruments, now being offered to the profession, and judge from examination that it is well calculated at least to hold the prolapsed uterus in its natural position. It is a beautifully made instrument, composed wholly of pure silver, and constructed upon correct mechanical principles. In cases which require mechanical support we believe it better adapted to that end than any other form of pessary, and would recommend its trial. In this connection, however, we would caution against the use of such instruments in all cases where they can be dispensed with, since foreign substances introduced into the vagina for the purpose of supporting the womb, are very apt to be productive of injury than otherwise.

